# The Role of Patiromer in Delaying the Onset of Renal Replacement Therapy in Patients with Advanced Renal Failure

**DOI:** 10.1155/2021/6987456

**Published:** 2021-09-07

**Authors:** Nand K. Wadhwa, Jason A. Kline, Sreedhar R. Adapa

**Affiliations:** ^1^Nephrology Division, NY Health, Stony Brook, NY, USA; ^2^Cooper Medical School of Rowan University, Camden, NJ, USA; ^3^The Nephrology Group, Inc., Visalia, CA, USA

## Abstract

Patients with chronic kidney disease (CKD) are at an increased risk of developing hyperkalemia, which can be potentially life threatening. Hyperkalemia is frequently encountered with renin-angiotensin-aldosterone system inhibitor (RAASi) therapy use in patients with CKD and often results in the underdosing or discontinuation of these drugs. RAASi therapy has been proven to delay the progression of CKD, ameliorate proteinuria, and reduce the overall risk of cardiovascular morbidity and mortality. Patiromer is a sodium-free, potassium-binding polymer used for the treatment of hyperkalemia. We present a case series of four patients with Stage 4 or 5 CKD in whom the initiation of dialysis was delayed with the use of patiromer. For one patient, dialysis was delayed by 18 months, whereas the remaining three patients, in whom hyperkalemia was one of the main complications, remain dialysis independent to date.

## 1. Introduction

Patients with chronic kidney disease (CKD) are at an increased risk of developing hyperkalemia because of decreased luminal secretion of potassium (K^+^) in the distal tubule [1]. Hyperkalemia is a potentially life-threatening electrolyte abnormality that can cause arrhythmias and cardiac arrest [1]. The main risk factors for developing hyperkalemia include decreased estimated glomerular filtration rate (eGFR), use of renin-angiotensin-aldosterone system inhibitors (RAASi), older age, male gender, diabetes mellitus, and metabolic acidosis [1, 2].

Treatment with RAASi has been proven to delay the progression of CKD, improve proteinuria, and decrease the overall risk of cardiovascular morbidity and mortality and is therefore recommended as a first-line treatment for hypertension in patients with CKD [3]. However, hyperkalemia is a frequent problem encountered with RAASi therapy in patients with CKD and often results in the underdosing or discontinuation of these drugs [4].

We present a case series of four patients with advanced Stage 4 or 5 CKD in which the initiation of dialysis was delayed following the use of the sodium-free potassium-binding polymer, patiromer. For one patient, initiation of dialysis was delayed by 18 months, whereas the remaining three patients are still dialysis independent. In all four patients, hyperkalemia was managed successfully with patiromer, despite the concomitant use of RAASi therapy.

## 2. Case Series

### 2.1. Patient 1

An 80-year-old woman was followed up in the outpatient nephrology clinic for the management of Stage 4 CKD and proteinuria. Other significant medical history included type 2 diabetes (T2D), hypertension, obesity, and heart failure with reduced ejection fraction (HFrEF). Her medications included irbesartan 150 mg daily, carvedilol 25 mg twice daily, torsemide 40 mg daily, and insulin. The patient's CKD and proteinuria were secondary to diabetes and had been diagnosed 5 years previously. Proteinuria was in the subnephrotic range, with a urine albumin-to-creatinine ratio (ACR) of 113–169.5 mg/mmol. She had a serum creatinine level of 221 *μ*mol/L and an eGFR of 20–25 mL/min/1.73 m^2^.

The dose of irbesartan was increased to reduce proteinuria, conserve renal function, and maximize the benefit of RAASi therapy for the management of her CHF. When irbesartan was increased to its maximum dose of 300 mg daily, K^+^ levels increased from 4.5 mmol/L to 5.7 mmol/L. The K^+^ level remained elevated between 5.5 mmol/L and 6.0 mmol/L on repeated testing. Patiromer 8.4 g/day was subsequently initiated, enabling the patient to continue taking the maximum dose of irbesartan (300 mg daily) without recurrence of hyperkalemia. Within a year, her urine ACR had decreased to 79.1 mg/mmol and showed further improvement, remaining around 11.3–22.6 mg/mmol.

The patient subsequently developed metastatic lung cancer and chose to spend her remaining days at home with family and under the care of home hospice services. She opted to withdraw from the management of her chronic medical conditions but remained on patiromer 8.4 g daily, with control of hyperkalemia for 3 years after the initiation of therapy. Renal function had been unchanged from the initial baseline visit when irbesartan was initially maximized, and proteinuria had remained controlled. The patient's CHF had remained stable with no HF-related hospital admissions while receiving the maximum dose of irbesartan (300 mg daily). Her serum potassium was 4.6, sodium 137 mmol/L, chloride 96 mmol/L, bicarbonate 24 mmol/L, calcium 9.3 mg/dL, phosphorus 4.3 mg/dL, and Mg 2.0 mg/dL during patiromer therapy. There were no reported complications with patiromer, such as constipation or other gastrointestinal side effects, hypokalemia, or hypomagnesemia.

### 2.2. Patient 2

A 70-year-old man with Stage 5 CKD due to diffuse diabetic glomerulosclerosis on kidney biopsy was referred for evaluation to assess the need to initiate peritoneal dialysis. His medical history included liver transplantation for hepatitis C–induced cirrhosis and liver cancer (managed with cyclosporine and mycophenolic acid [MPA] for immunosuppression and ledipasvir/sofosbuvir therapy for hepatitis C). Other significant medical history included nephrotic-range proteinuria, hyperkalemia, HF with preserved ejection fraction with a prior bioprosthetic aortic valve replacement, coronary artery disease with stent placement, T2D (being treated with insulin), hypertension, and gout. His medications included torsemide 100 mg once daily, metolazone 5 mg once daily, carvedilol 25 mg twice daily, cyclosporine, MPA, insulin, and allopurinol. He was clinically hypervolemic due to volume overload but was not uremic. Evaluation of laboratory parameters showed the following: serum creatinine 562.4 *μ*mol/L, eGFR 9 mL/min/1.73 m^2^, urine ACR 463.6 mg/mmol, and serum K^+^ 5.0 mmol/L.

Peritoneal dialysis was not feasible because of his liver transplant. It was not possible to obtain arteriovenous (AV) fistula access because of the lack of veins in the upper extremities. It was decided to place the AV graft 2 weeks before initiating dialysis. To meet his fluid intake goals, the patient was advised to consume fruits and vegetables (4–5 servings per day) rather than only drinking water. He was also counselled to reduce his dietary intake of animal-based protein and instead consume more plant-based protein. Treatment with metolazone was discontinued, and the patient was prescribed torsemide 60 mg once daily and spironolactone 25 mg once daily (three times a week) after which his volume status improved and stabilized. The patient was also started on patiromer 8.4 g once daily (two to three times a week) in order to maintain his serum K^+^ levels below 5.6 mmol/L. After clinical evaluation, he was placed on the deceased donor kidney transplant list.

Evaluation of his laboratory parameters at subsequent follow-up appointments over 18 months showed that his eGFR remained stable at 8–10 mL/min/1.73 m^2^ and urine ACR decreased to 60.1 mg/mmol. Serum albumin remained between 42 and 45 g/L, whereas serum bicarbonate ranged from 20 to 23 mmol/L. Serum K^+^ fluctuated between 4.7 and 5.6 mmol/L. His serum sodium 140 mmol/L, chloride 95 mmol/L, calcium 8.8 mg/dL, phosphate 4.3 mg/dL, and magnesium 2.0 mg/dL were within normal range while receiving patiromer. His liver function also remained normal, and his glycated hemoglobin (HbA_1c_) ranged from 26.8 to 32.2 mmol/mol. After 18 months from the time he was supposed to start dialysis, he continued to remain clinically euvolemic with no signs and symptoms of uremia and was not on dialysis. There were no reports of adverse events related to patiromer. To date, he remains on the deceased donor kidney transplant list.

### 2.3. Patient 3

A 71-year-old man with Stage 5 CKD due to autosomal dominant polycystic kidney disease was referred for the evaluation to initiate home hemodialysis. His medical history included liver cysts, hypertension, nephrolithiasis, vitamin D deficiency, and hyperkalemia. He was on the deceased donor kidney transplant list at the time of his referral, at which time his prescribed medications included furosemide 80 mg daily, spironolactone 25 mg daily (twice a week), metoprolol succinate 50 mg daily, hydralazine 50 mg three times daily, and doxazosin 2 mg daily. He reported feeling well and had been actively pursuing his hobby of vintage car restoration, which involved frequent travel, so he questioned the need to start dialysis treatment. He was not clinically uremic and reported having a good appetite but was clinically euvolemic. Evaluation of laboratory parameters showed the following results: serum creatinine 683.5 *μ*mol/L, eGFR 7 mL/min/1.73 m^2^, and serum K^+^ 5.2 mmol/L. His 24-hour urine protein was 53.9 mg/day. The left brachiocephalic AV fistula was revised to a left brachial basilic transposition due to the lack of maturation. In order to increase the likelihood of receiving a kidney transplant, his transplant status was updated to include the hepatitis C-positive deceased donor list. He was advised to increase his consumption of water to prevent further progression of kidney cysts and to reduce his sodium intake. In line with data presented in the recent Kidney Disease: Improving Global Outcomes (KDIGO) controversies conference report [5], he was advised to decrease his intake of animal-based protein and to increase plant-based protein intake instead and also to consume more fruits and vegetables (4–5 servings a day).

The patient was started on treatment with patiromer 8.4 g once daily (2-3 times a week) to maintain his serum K^+^ levels below 5.6 mmol/L. Evaluation of laboratory parameters at subsequent follow-up appointments showed that his eGFR remained between 6.5 and 7.5 mL/min/1.73 m^2^, serum K^+^ fluctuated between 4.9 and 5.8 mmol/L, and serum albumin remained at 43–47 g/L. His other electrolytes were serum sodium 145 mmol/L, chloride 100 mmol/L, bicarbonate 24, calcium 9.0 mg/dL, phosphate 5.7 mg/dL, and magnesium 2.3 mg/dL. His liver function remained normal. He was clinically euvolemic with no signs and symptoms of uremia and still reported having a good appetite. The patient was still not on dialysis 16 months after its proposed initiation and tolerated patiromer without adverse events. To date, he remains on the deceased donor kidney transplant list, including the hepatitis C–positive pool.

### 2.4. Patient 4

A 69-year-old man was followed up in the outpatient nephrology clinic for Stage 5 CKD and proteinuria. His medical history was significant for T2D, hypertension, hyperlipidemia, and diabetic neuropathy. His medications included pravastatin 20 mg daily, atenolol 50 mg daily, lisinopril 40 mg daily, gabapentin 300 mg daily, and insulin. His serum creatinine was 343.1 *μ*mol/L with an eGFR of 15 mL/min/1.73 m^2^. The patient had subnephrotic-range proteinuria with a urine ACR of 61.8 mg/mmol. Serum K^+^ levels ranged from 5.3 to 6.0 mmol/L, even after repeated dietary counseling. The patient worked as a farm laborer, and his diet included predominantly fruits and vegetables, which are rich in K^+^.

The patient was started on patiromer 8.4 g once daily and serum K^+^ levels subsequently trended within the normal range (3.5–5.1 mmol/L). The other serum electrolytes remained within a normal range, including sodium 136 mmol/L, chloride 104 mml/L, bicarbonate 23 mmol/L, calcium 9.1 mg/dL, phosphorus 4.1 mg/dL, and magnesium 1.8 mg/dL. Proteinuria and renal function remained stable for almost 12 months, and he continued to take lisinopril 40 mg daily with no interruptions. Hyperkalemia recurred once during this period after the patient stopped taking patiromer. He received education on his medication including diet and was restarted on patiromer. No episodes of hypercalcemia noted when he was taking patiromer. Over time, his eGFR declined to 9–12 mL/min/1.72 m^2^, but there was no recurrence of hyperkalemia. No gastrointestinal side effects or hypokalemia was encountered during patiromer therapy. After 18 months of follow-up, dialysis was initiated for the management of uremic symptoms.

## 3. Discussion

Hyperkalemia is one of the relatively common complications encountered in patients with impaired renal function. The typical Western diet contains an average daily K^+^ intake of ∼2600 mg [6]. The role of the kidney in maintaining K^+^ homeostasis is critical [7]: it is the primary organ responsible for maintaining total body K^+^ by excreting 90% of K^+^ intake. The gastrointestinal tract (primarily the colon) accounts for 10% of K^+^ secretion [8]. In individuals with normal kidney function, the proximal tubule and loop of Henle reabsorb 80–90% of the filtered K^+^ load, and luminal secretion in the distal tubule constitutes the primary mechanism of renal K^+^ excretion [1]. However, this luminal secretion of the K^+^ is impaired in CKD, resulting in hyperkalemia, which can be exacerbated by RAASi treatment.

As demonstrated by the African American Study of Kidney Disease and Hypertension (AASK) trial, conducted in 1,094 nondiabetic African American patients with hypertensive CKD [9], the occurrence of hyperkalemic episodes is higher when GFR is <30 mL/min/1.73 m^2^ than when GFR is >50 mL/min/1.73 m^2^. In contrast to the AASK trial, a higher event rate of hyperkalemia was reported by Einhorn et al. [10] in their analysis of the United States Veterans Health study. This was due to the inclusion of patients with diabetes and of a higher proportion of elderly subjects and those with multiple comorbidities [10]. The magnitude and frequency of hyperkalemia varies depending on a multitude of factors. In particular, several studies have shown the risk of hyperkalemia increases in CKD with the use of RAASi therapy [9, 11]. Hyperkalemia can cause potentially life-threatening cardiac arrhythmias and is associated with an increased risk of major cardiovascular morbidity and mortality in various populations [12, 13].

The incidence of hyperkalemia in patients with CKD receiving dual-agent RAASi therapy is approximately 5–10%, compared with <2% in patients without CKD on RAASi monotherapy [14]. The most common approach to reduce the risk of hyperkalemia is to downtitrate the dose or discontinue the use of RAASi [4]. However, suboptimal dosing of RAASi is associated with adverse outcomes, including the increased risk of cardiac events, mortality, and earlier progression to end-stage renal disease, which in turn leads to increased health care costs [15].

Various clinical practice guidelines recommend the use of RAASi therapy in adults for the management of common comorbidities. Treatment with angiotensin-converting enzyme inhibitors (ACEi) or angiotensin II receptor blockers (ARBs) prevents the progression of CKD in both people with and without diabetes and reduces urine albumin excretion [16]. Treatment with ACEi or ARBs also improves renal outcomes in patients with hypertension and CKD [17]. Furthermore, ACEi and ARBs have been shown to reduce morbidity and mortality in patients with HFrEF and also prevent symptomatic HF [18]. Treatment with mineralocorticoid receptor antagonists (MRAs) decrease mortality and morbidity in patients with New York Heart Association (NYHA) functional Class II–IV HFrEF [18]. Given the benefits of RAASi therapy in reducing the risk of cardiovascular events and CKD progression, clinicians are commonly faced with the challenge of hyperkalemia in CKD when using RAASi.

Health benefits associated with foods enriched in K^+^ are their alkalinity and fiber, micronutrient (vitamin and mineral, including K^+^), and phytochemical content [19, 20]. A diet rich in K^+^ reduces the risk of cardiovascular disease, stroke, nephrolithiasis, and cholesterol and improves the acid-base balance, blood pressure, and progression of chronic kidney disease, thereby reducing mortality rate [21, 22]. Patients with advanced CKD are at higher risk of developing hyperkalemia with a K^+^-liberalized diet. The relationship between dietary K^+^ intake and serum K^+^ levels is not well established, although studies report that the restriction of dietary K^+^ intake has minimal impact on reducing serum K^+^ levels [4]. Recently, a KDIGO controversies conference report suggested that plant-based diets may be more suitable for patients with CKD and that they should be educated on the benefits of low-K^+^ plant-based foods, particularly vegetables that can be incorporated into their diet [5]. The use of loop diuretics, either alone or in conjunction with thiazide diuretics, will aid in the urinary excretion of K^+^ by increasing flow and sodium delivery to the distal nephron [14]. However, in patients with advanced CKD, diuretic therapy for the management of hyperkalemia can be inadequate [14].

Oral K^+^-binding therapies can enable and facilitate the appropriate use of a K^+^-rich diet and of RAASi in patients with CKD, CHF, or both. Oral K^+^-binding agents like sodium polystyrene sulfonate (SPS), sodium zirconium cyclosilicate (SZC), and patiromer are approved by the United States Food and Drug Administration (FDA) for the treatment of hyperkalemia [1]. These agents work by exchanging cations for K^+^ in the gastrointestinal (GI) tract, resulting in K^+^ binding and the excretion of K^+^ in the feces. These K^+^-binding agents should not be used for emergency treatment of life-threatening hyperkalemia because of their delayed onset of action.

SPS is a sodium-K^+^ exchange resin that was approved by the FDA in 1958 for the treatment of hyperkalemia [4]. However, robust clinical safety and efficacy data for SPS in hyperkalemia were lacking prior to the FDA approval. With its foul taste and consistency, and the potential risk for serious gastrointestinal injury [23], it has not been demonstrated to be an effective approach in long-term clinical studies to manage chronic hyperkalemia.

The K^+^-lowering efficacy and safety profiles of the two newer K^+^-binding agents, SZC and patiromer, were evaluated in phase 2 and 3 randomized controlled trials prior to their approval for use in clinical practice [4].

SZC is an inorganic, insoluble, and highly selective K^+^ binder approved in the United States and European Union for the treatment of hyperkalemia in adults [24]. SZC binds K^+^ and ammonium ions in GI tract in exchange for sodium and hydrogen ions, and the bound K^+^ is subsequently excreted in the feces [25, 26]. Clinical studies in hyperkalemic patients have shown that most individuals treated with SZC achieve normal serum K^+^ levels within 4 hours of their first 10 g dose and that normokalemia can be maintained for up to 28 days with once daily doses of 5–15 g [27, 28]. Furthermore, the tolerability profile of SZC has been shown to be comparable with that of placebo [27, 28]. Increases in serum bicarbonate have been observed during SZC treatment [25, 28], potentially due to SZC binding ammonium ions [26]. SZC should not be used as emergency treatment for life-threatening hyperkalemia because of its delayed onset of action.

Spinowitz et al. [24] evaluated the use of SZC for the control of hyperkalemia over 1 year in a study that included 746 participants with hyperkalemia, of whom the majority had an eGFR below 60 mL/min/1.73 m^2^ (74%) and were receiving RAASi therapy (65%). During the 12-month maintenance phase, SZC was administered once daily dissolved in a small amount of water, taken separately from other medications by at least 2 hours. Overall, SZC was well tolerated, and common adverse events that occurred during treatment included constipation (6%), hypertension (11%), and peripheral edema (10%) [24]. In a one-year study of SZC, edema events occurred more frequently in the subgroup of patients with eGFR of <30 mL/min/1.73 m^2^ compared with those with eGFR of ≥30 mL/min/1.73 m^2^ (21% vs. 11%, respectively) [29]; SZC exchanges sodium for K^+^, which may explain the occurrence of hypertension and edema with this medication, especially if higher doses are used. During months 3–12 of the maintenance phase, the serum K^+^ level was maintained at ≤5.1 mmol/L in 88% of participants and ≤5.5 mmol/L in 99% of participants [24].

Patiromer is a sodium-free, K^+^-binding polymer that exchanges calcium for K^+^ in the GI tract, predominantly in the colon [2]. It is an oral suspension of spherical beads of uniform size (∼100 *μ*m) that are too large to be absorbed passively in the GI tract [30]. The drug substance consists of the active moiety, patiromer, and the calcium–sorbitol complex, the counterion that acts in K^+^ exchange, resulting in the binding of K^+^ and its fecal excretion [2].

Patiromer has a delayed onset of action of 4–7 hours, so it should not replace existing emergency treatments for life-threating hyperkalemia [2]. The initial starting dose of patiromer is 8.4 g once daily, and the dose can be titrated as necessary at intervals of 1 week or longer, in increments of 8.4 g, up to a maximum dose of 25.2 g once daily [2]. No dosage adjustments are needed for patients on dialysis or with renal impairment [2]. Clinical drug–drug interaction studies of patiromer conducted in healthy volunteers showed that 9 out of 12 medications commonly used in the CKD population did not demonstrate clinically relevant drug–drug interactions when administered together with patiromer [31]. Nonetheless, it is recommended that administration of concomitant medications should occur at least 3 hours before or after administration of patiromer [31].

In the patient cases reported here, hyperkalemia treatment with patiromer was selected for several reasons. Patiromer uses calcium rather than sodium as the exchange ion, thus the risk of hypertension and edema, particularly in patients who should restrict their sodium intake or are prone to fluid overload, such as those with heart failure or hypertension, is avoided. The choice of a binder that does not contain sodium appears to be particularly important in the patient with volume overload, and the patient who is advised to restrict sodium intake.

In an exploratory analysis of phase 3 OPAL-HK study (4-week initial treatment phase of 243 patients; 8-week randomized withdrawal phase of 107 patients), patiromer was associated with a reduction in aldosterone levels independent of plasma renin activity in patients with CKD and hyperkalemia on RAAS inhibitors [32]. In addition, patiromer use was also associated with decreased systolic and diastolic blood pressure, as well as albuminuria [32]. Pharmacodynamic effects of patiromer in an animal model with chronic hyperkalemia showed a significant reduction in serum K^+^ that correlated with serum aldosterone [33]. The effect of patiromer on aldosterone may be related indirectly to reduction in serum K^+^ level but remains unexplained. Furthermore, the patients with CKD Stage 4 or 5 were being managed with RAAS inhibitors, either with maximally dosed ACEi or an ARB or with the mineralocorticoid receptor antagonist, spironolactone. Placebo-controlled clinical trial data suggest that treatment of hyperkalemia with patiromer may maintain RAASi therapy by reducing the risk of recurrent hyperkalemia [34] and that patiromer may prevent the development of hyperkalemia in patients initiating spironolactone, thereby allowing uptitration to optimal doses [35, 36]. More recently, the AMBER trial in patients with resistant hypertension and advanced CKD [35] showed that patiromer treatment was more effective than placebo at maintaining spironolactone treatment over 12 weeks, resulting in a statistically larger mean cumulative dose of spironolactone. A Phase-3b, multinational, double-blind, placebo-controlled, withdrawal study (DIAMOND; NCT03888066) is currently underway in HFrEF patients who develop hyperkalemia while receiving RAASi therapies, in order to evaluate the effects of patiromer compared with placebo on changes in serum K^+^ levels (primary end point) and hyperkalemia events, durable enablement of MRA target dose, and hyperkalemia-related hard outcomes(key secondary end points) [37].

Finally, patiromer is an appropriate choice of treatment in the cases described here, based on its rigorous safety and tolerability profile, with data on up to 52 weeks of exposure [38]. The most common side effects with patiromer are typically related to GI, such as constipation and diarrhea [2, 39]. Because patiromer has the potential to bind magnesium in the GI tract, it is recommended that clinicians monitor magnesium in patients receiving patiromer and consider an increased dietary magnesium intake or magnesium supplementation in patients who develop low serum magnesium levels. In clinical trials, 9% of patients developed a serum magnesium level below 1.4 mg/dL, and none developed a level below 1.0 mg/dL [2]. Because patiromer uses calcium as the counterexchange ion, some of the released calcium appears to bind to intestinal phosphate. In patients with hyperphosphatemia, this binding may result in reductions in serum and urine phosphate [40]. Very rarely, cases of hypercalcemia have been reported [41]. Of note, a recent 4-year pharmacovigilance report indicates that patiromer's adverse event profile in the real-world setting is predictable and consistent with clinical trial data, with no evidence of any new safety signals to date [42].

## 4. Conclusion

The suboptimal dosing of RAASi secondary to hyperkalemia results in detrimental outcomes in the patients with heart failure and CKD and is associated with significant economic burden. Hyperkalemia is one of the major complications in CKD and limits complete RAAS blockade. A K^+^-exchange polymer, such as patiromer, is effective in controlling hyperkalemia and maintaining normokalemia, while, at the same time, potentially maximizing the benefits of RAASi therapy and an increased consumption of a healthy plant-based diet. In the current case series, we show that patiromer was effective in controlling hyperkalemia in conjunction with dietary counselling and thereby delaying the initiation of dialysis in four patients with Stage 4 or 5 CKD. This strategy requires further study in a controlled setting.

## Data Availability

No data were used to support this study.
